# The CLUES study: a cluster randomized clinical trial for the evaluation of cardiovascular guideline implementation in primary care

**DOI:** 10.1186/1472-6963-13-438

**Published:** 2013-10-24

**Authors:** Arritxu Etxeberria, Itziar Pérez, Idoia Alcorta, Jose Ignacio Emparanza, Elena Ruiz de Velasco, Maria Teresa Iglesias, Domingo Orozco-Beltrán, Rafael Rotaeche

**Affiliations:** 1 Hernani Health Center, Gipuzkoa Health District, Basque Health Service, c/Aristizabal 1, 20120 Hernani, Spain; 2 Bidasoa Integrated Healthcare Organization, Basque Health Service, Irun, Spain; 3 University Hospital, IIS Biodonostia, San Sebastian, Spain; 4 Bilbao Health District, Basque Health Service, Bilbao, Spain; 5 Miguel Hernandez University, Alicante, Spain; 6 Alza Health Centre, Basque Health Service, San Sebastian, Spain

**Keywords:** Diabetes, Education, Medical continuing, Guidelines, Health plan implementation, Hyperlipidemias, Hypertension, Primary health care, Risk factors, Cardiovascular

## Abstract

**Background:**

The appropriate care for people with cardiovascular risk factors can reduce morbidity and mortality. One strategy for improving the care for these patients involves the implementation of evidence-based guidelines. To date, little research concerning the impact of such implementation strategies in our setting has been published. Aims. To evaluate the effectiveness of a multifaceted tailored intervention in the implementation of three cardiovascular risk-related guidelines (hypertension, type 2 diabetes and dyslipidemia) in primary care in the Basque Health Service compared with usual implementation.

**Methods/Design:**

A two-year cluster randomized clinical trial in primary care in two districts in the Basque Health Service. All primary care units are randomized. Data from all patients with diabetes, hypertension and those susceptible to coronary risk screening will be analyzed.

Interventions. The control group will receive standard implementation. The experimental group will receive a multifaceted tailored implementation strategy, including a specific web page and workshops for family physicians and nurses.

Endpoints. Primary endpoints: annual request for glycosylated hemoglobin, basic laboratory tests for hypertension, cardiovascular risk screening (women between 45–74 and men between 40–74 years old). Secondary endpoints: other process and clinical guideline indicators.

Analysis: Data will be extracted from centralized computerized medical records. Analysis will be performed at a primary care unit level weighted by cluster size.

**Discussion:**

The main contribution of our study is that it seeks to identify an effective strategy for cardiovascular guideline implementation in primary care in our setting.

**Trial registration:**

Current Controlled Trials, ISRCTN88876909

## Background

### Diabetes and cardiovascular risk factors

High blood pressure, dyslipidemia and diabetes are the main clinical conditions presented by patients with multiple morbidities [1].

Cardiovascular disease is the leading cause of death in both the Basque Country and Spain as a whole. Despite this, cardiovascular disease-related mortality has decreased over the past few years due, amongst other reasons, to the better diagnosis and treatment of cardiovascular risk factors [2].

Current data suggest that there remains significant room for improvement in cardiovascular risk factor and diabetes care in our setting. According to data from the Provider Agreement in 2008 only 41.3% of diabetic patients in the Autonomous Community of the Basque Country had a glycosylated hemoglobin (HbA1c) level of less than 7.5%, basic analysis was performed in 44.9% of patients and only 27% had a blood pressure of less than 140/80 mm Hg.

The almost anecdotal use of tools to calculate cardiovascular risk at that time suggests that statin treatment in primary prevention is chosen on the basis of cholesterol level rather than overall cardiovascular risk [3].

As far as hypertensive patients in our setting are concerned, it is estimated that only 33% have their blood pressure well controlled and that 46.4% do not receive the antihypertensive agent of choice [4].

### Clinical practice guidelines as a tool for improving healthcare

The implementation of evidence-based clinical practice guidelines (CPGs) may help to ensure that the care of patients with cardiovascular risk factors or diabetes complies with the best quality criteria and standards.

CPGs are statements that include recommendations intended to optimize patient care which are informed by a systematic review of evidence and an assessment of the benefits and harms of alternative care options [5]. As such, they have the potential to reduce variability and improve healthcare [6].

In the past few years we have witnessed the consolidation of a CPG program in both the Basque Country and the Spanish National Health System as a whole that has led to the Guiasalud program [7], the development of a common methodology for drafting such guidelines [8] and the availability of an increasing number of guidelines prepared in Spain.

Thus, in the cardiovascular field, 2008 and 2009 saw the publication of the “Clinical practice guideline on the management of lipids as a cardiovascular risk factor” [9], an update to the regional “Clinical practice guideline on arterial hypertension” [4] and the “Clinical practice guideline on type 2 diabetes” [10], the latter of which was drafted within the framework of the National Health System's Clinical Practice Guideline Program in collaboration with the Health Technology Assessment Agency Osteba. These three guidelines are available from Guiasalud [7] and have been included in the National Guideline Clearinghouse.

### Design of CPG implementation strategies

The publication and dissemination of a CPG does not, however, ensure its application in clinical practice, therefore effective and viable implementation plans for the organizational context at which it is aimed must be designed. Implementation must be considered to be a planned process whose main characteristics are dynamism and uniqueness [6].

It is important that guideline implementation interventions are designed in accordance with a coherent theoretical foundation, a body of evidence to support them and taking into account the barriers and facilitators of the local setting [6,11-13].

#### ***Theoretical foundation***

Grol’s 10-step model [11] is a theoretical reference for guideline implementation [6]. This model brings together elements from different disciplines, such as the diffusion of innovation theory [14], the reasoned action theory [15] and social cognitive theory [16], and postulates that professionals pass through different phases or stages (orientation, insight, acceptance, change and maintenance) when achieving a change, with specific barriers predominating in each phase. This study concentrates on the orientation, insight and acceptance phases.

#### ***Barriers and facilitators***

Although there are numerous studies concerning the barriers and facilitators for CPG implementation [17], this is not the case in our setting. As a result, before designing the guideline implementation intervention, we undertook a study to explore the barriers faced by CPGs in primary care using the Delphi technique [18]. The main barriers detected are the following [18].

Dimension: Presentation of the guidelines**:**

– The teaching method used by the speakers at each session.

– The choice of speaker.

Dimension: Format of the guidelines:

– The need for a summarized version.

– The need to stimulate and promote the on-line version, which makes term search easier, and the links within the guidelines and to other material of interest.

– The need to enhance user participation (discussion fora, asking questions, debates, etc.).

Dimension: Use and utility facilitators:

– Application of CPGs gives good results in clinical practice.

– The need to attach action protocols or other practical tools to the guidelines.

Internal barriers:

– Willingness of the physicians themselves.

– Too much time and effort required to understand the CPGs.

– Lack of acceptance of guidelines as a work tool.

– Discouragement due to lack of use at other healthcare levels.

External barriers:

– Specialized practice does not follow the guidelines.

– Lack of dissemination and implementation in specialized care.

– Pressure from the pharmaceutical industry.

– Methodology followed to learn the guidelines.

#### ***Effectiveness of guideline implementation interventions***

According to Grimshaw’s systematic review [12], the effectiveness of different clinical practice guideline dissemination and implementation strategies varies but, in general, tends to be modest. The dissemination of educational materials tends to be poorly effective but at low-cost, whereas continual medical training is more effective if undertaken in small groups using realistic scenarios [19-21]. The efficacy of opinion leader-based training tends to vary [12]. Multifaceted interventions appear to make sense if they are intended to overcome specific barriers [19]. Indeed, tailored interventions are more effective than passive guideline dissemination [13].

In the field of diabetes, the review by Shojania [22] showed that multifaceted interventions provided a larger effect than interventions involving a single component. Educational interventions showed an acceptable efficacy. In the field of hypertension, educational interventions aimed at healthcare professionals resulted in a modest improvement in blood pressure control [23]. The most effective strategy for implementing the use of risk tables in cardiovascular disease prevention is still unknown [24].

#### ***Context***

The intervention and study were designed in the context of a primary care research group with a clinical and/or methodological profile involved in the drafting and implementation of evidence-based guidelines. The intervention was aimed at primary care professionals (physicians and nurses) and no organisational changes or interventions in patients were considered. A further assumption was that the proposed interventions had to be feasible and result in a reasonable cost to the health system.

As no single implementation strategy is effective in all contexts, the impact of such strategies needs to be evaluated by way of studies with a robust design and low risk of bias. Randomized designs are the gold-standard for assessing healthcare interventions [12,25].

Although we are witnessing significant support for the drafting of CPGs by public health organisations in Spain, evaluation of their subsequent implementation remains uncommon [26].

In our setting, the availability of three recently published CPGs in the cardiovascular field provides a unique opportunity to assess CPG implementation strategies in primary care.

### Main endpoint

To evaluate the effectiveness of a multifaceted tailored intervention in the implementation of three cardiovascular risk-related CPGs (hypertension, type 2 diabetes and lipids as cardiovascular risk factor) in primary care in the Basque Health Service compared with usual implementation.

### Secondary endpoints

• To evaluate the impact of the intervention on the degree of compliance with process quality indicators and subrogate clinical endpoints for the care of patients with type 2 diabetes.

• To evaluate the impact of the intervention on the degree of compliance with process quality indicators and subrogate clinical endpoints for the care of patients with hypertension.

• To evaluate the impact of the intervention on the degree of compliance with coronary risk screening process quality indicators.

• To evaluate the impact of the intervention on the prescription of statins in primary and secondary prevention.

## Methods/Design

### Study design and setting

This study is a cluster randomized trial conducted in two urban primary care districts in the Basque Health Service (Ekialde and Bilbao). These districts cover 36.9% of the population in the Basque Country. Primary care units (PCU) will be randomly assigned.

A cluster-type design is selected as the intervention was aimed at professionals working in a PCU and due to the risk of contamination between professionals working in the same PCU [27,28].

**Figure 1:**
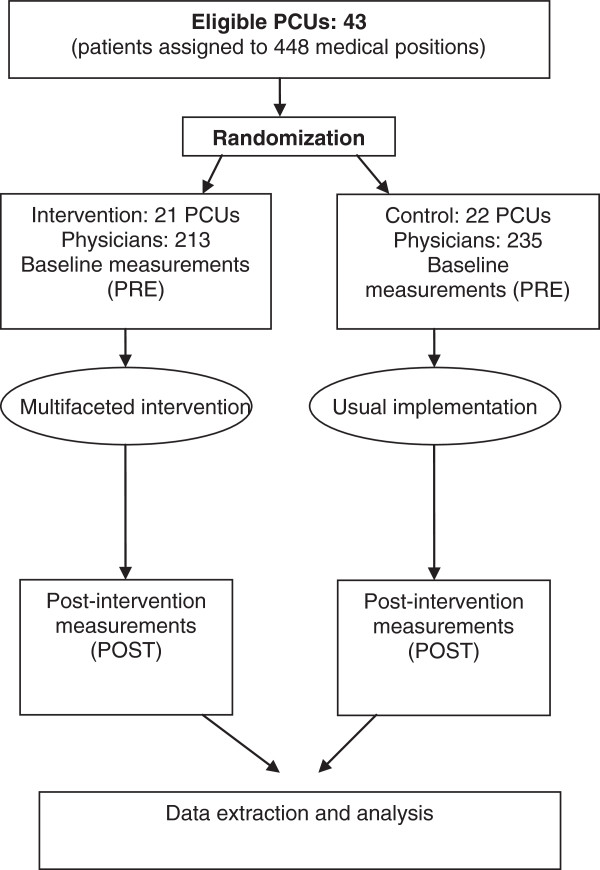
Study design.

A repeated cross-sectional design is used for all patients who attend during the baseline period (pre-intervention, or “PRE”) and in the post-intervention period (“POST”), with different samples. Such an approach is appropriate when the aim of the study is to determine the impact of CPG implementation on the population and provides greater long-term power [27,28]. It also allows minor baseline differences that may arise due to cluster randomization to be compensated. The study design is shown in Figure 1.

### Study population

Consent to participate was received from the heads of all PCUs from both districts, therefore they were all randomized (43 PCUs). All professionals (physicians and nurses) appointed to medical jobs in family medicine who agreed to participate in the study were included. Data from all patients who attend their PCU during the study period (PRE and POST intervention) and assigned to the PCUs and physicians/nurses who participate in the study will be analyzed, if they complied with one of the following inclusion criteria:

– A diagnosis of type 2 diabetes in the medical record.

– A diagnosis of hypertension in the medical record.

– Population susceptible to cardiovascular risk screening: women aged 45–74 years and men aged 40–74 years with no ischemic heart disease.

– Patients who commenced statin treatment during the study period.

– Patients diagnosed with ischemic heart disease during the study period.

The exclusion criteria are: patients assigned physicians or nurses who declined to participate in the study, younger than 14 years of age, patients assigned to physicians occupying two or more medical posts belonging to the intervention and control groups at the same time, and patients who don’t attend their PCU during the study period.

### Interventions

The control group will receive usual implementation, namely mailing of the guideline, publication in the intranet and presentation sessions in the PCU. These sessions will be led by physicians trained by the trainers responsible for teaching the intervention group.

In addition to the control group interventions, the multifaceted implementation for the experimental group also includes:

– Identical presentation sessions to those for the control group but given by physicians who took part in the guideline development process.

– The design of a specific web page with the guideline recommendations aimed at the action, with quick access, application tools (algorithms and tables with links to the main recommendations, patient materials), the possibility to ask questions, links to drug-related information.

– Three-hour workshops for family physicians and nurses, eight cardiovascular risk workshops for family physicians and a further eight for nursing staff, and four diabetic foot workshops for nursing staff. These workshops will be mostly led by the physicians responsible for the guidelines with the colaboration of two non-involved nurses.

### Endpoints

#### ***Primary endpoints***

– Diabetes: percentage of patients with annual request for glycosylated haemoglobin (HbA1c).

– Hypertension: percentage of patients with annual request for basic analysis, including albumin-creatinine ratio.

– Dyslipidemia: males between 40–74 and females between 45–74 years with at least one cardiovascular risk assessment (except males and females with cardiovascular disease).

#### ***Secondary endpoints***

– Diabetes: percentage of patients with HbA1c lower than 7%, percentage of patients with blood pressure lower than 140/80 mm Hg, percentage of patients with confirmed basic analysis, percentage of patients with confirmed cardiovascular risk assessment, percentage of patients with confirmed foot examination and new pharmacological treatment started with metformin.

– Hypertension: percentage of non-diabetic patients with blood pressure lower than 140/90 mm Hg, percentage of patients with annual cardiovascular risk assessment, percentage of patients starting pharmacological treatment with diuretics, beta-blockers or angiotensin-II receptor blockers.

– Dyslipidemia: percentage of patients aged between 35 and 74 years with no cardiovascular disease starting a statin treatment with previous cardiovascular risk assessment, new statin treatments in women aged over 35 years with no cardiovascular disease or diabetes, percentage of patients with a new diagnosis of coronary heart disease receiving statin treatment.

### Sample size

The following aspects were taken into account when calculating the sample size:

• On average, 45-50% of diabetic patients assigned to a healthcare professional undergo at least two annual HbA1c analyses (standard deviation 15-20%). An annual analysis is requested in around 35% of hypertensive patients. (Data taken from the Provider Agreement, 2007).

• From a clinical and operational viewpoint, an intervention strategy would be worthwhile if these averages could be increased to 55-60% for diabetes and 43% for hypertension.

• The size of the PCUs is similar, with around 10 physicians per PCU, and the number of patients assigned to each clinician is also similar.

• The intracluster correlation coefficient (ICC) is 0.10 [29-31].

In light of these considerations, approximately 100 physicians per group (10 PCUs in each group) would be needed for an α of 0.05 and a β of 0.20. Applying the cluster-related design effect gives a final number of 20 PCUs per group (intervention and control).

### Randomization

Allocation is performed by clusters. PCUs are assigned to the intervention or control group following a computer-generated randomization sequence. Randomization is performed centrally by a researcher not involved in the study who was blind to the identity of the PCU.

### Masking

Professionals implementing the intervention are not blinded to the assignment group. However, data extraction will be performed centrally by computer technicians not involved in the study and data will be treated by research personnel after anonymisation.

### Analysis

Data will be extracted from centralized computerized medical records. Analysis will be performed at the PCU level taking the cluster design into account [32]. Indicators will be obtained for each PCU before and after the intervention and the differences will be weighted by cluster size. All analyses will be performed using Student’s *t*-test as implemented in SPSS 19. A secondary multi-level analysis will be performed using MLwiN (version 2.21) to determine the intracluster correlation coefficients (ICCs).

Analysis will be performed on an intention-to-treat basis.

### Ethical considerations

This trial was approved by the Clinical Research Ethics Committee of the Basque Country. The protocol was registered in the Current Controlled Trials database (ISRCTN 88876909). Funding was provided by the Spanish Ministry of Health as part of the 2007–2008 collaboration agreement anticipated in the National Health System’s quality plan between the Carlos III Research Institute, an autonomous body that forms part of the Spanish Ministry of Science and Innovation, and the Basque Government’s Department for Health and Consumer Affairs (OSTEBA).

## Discussion

There is a growing need to identify effective implementation strategies for CPGs for the management of cardiovascular risk factors in primary care. This study is one of the few cluster randomized trials concerning the implementation of clinical practice guidelines in Spanish primary care.

This study focuses on multiple cardiovascular risk factors as primary endpoints. Its pragmatic design is aimed at determined the actual impact of implementing CPGs for type 2 diabetes, hypertension and dyslipidemia via a multifaceted intervention based on identifying local barriers to CPG implementation, and it is aimed at primary care professionals.

The limitations of this study include the use of medical records as data source. Giving the nature of the study, the intervention cannot be masked.

The findings of this study will allow a more effective CPG planning in the primary cardiovascular care field to be designed. Similarly, it will allow the key aspects of recommendation implementation in our setting to be identified. All this information may prove useful in the organisational transformation currently underway in the Basque Country to adapt the health system to the care needs of patients with chronic diseases.

## Abbreviations

CPG: Clinical Practice Guideline; HbA1c: glycosylated hemoglobin; ICC: Intracluster Correlation Coefficient; PCU: Primary Care Unit.

## Competing interests

The authors declare that they have no competing interests.

## Authors’ contributions

RR, AE, IP, IA designed and planned the study. DO gave advice concerning the study design. JIE calculated the sample size and helped to design the study. JIE and MI planned the statistical analysis. ERV helped to plan the study and the intervention. AE drafted the study protocol. All authors reviewed the draft version, made suggestions and approved the final version.

## Pre-publication history

The pre-publication history for this paper can be accessed here:

http://www.biomedcentral.com/1472-6963/13/438/prepub
